# Evaluation of Mg Compounds as Coating Materials in Mg Batteries

**DOI:** 10.3389/fchem.2019.00024

**Published:** 2019-01-30

**Authors:** Tina Chen, Gerbrand Ceder, Gopalakrishnan Sai Gautam, Pieremanuele Canepa

**Affiliations:** ^1^Department of Materials Science and Engineering, University of California, Berkeley, Berkeley, CA, United States; ^2^Materials Science Division, Lawrence Berkeley National Laboratory, Berkeley, CA, United States; ^3^Department of Mechanical and Aerospace Engineering, Princeton University, Princeton, NJ, United States; ^4^Department of Materials Science and Engineering, National University of Singapore, Singapore, Singapore

**Keywords:** Mg batteries, first-principles calculation, density functional theory, coating materials, intercalation batteries, solid electrolytes, multivalent ion batteries

## Abstract

Mg batteries utilizing a Mg metal anode with a high-voltage intercalation cathode define a potential pathway toward energy storage with high energy density. However, the making of Mg batteries is plagued by the instability of existing electrolytes against the Mg-metal anode and high-voltage cathode materials. One viable solution to this problem is the identification of protective coating materials that could effectively separate the distinct chemistries of the metal-anode and the cathode materials from the electrolyte. Using first-principles calculations we mapped the electrochemical stability windows for non-redox-active Mg binary and ternary compounds in order to identify potential coating materials for Mg batteries. Our results identify Mg-halides and Mg(BH_4_)_2_ as promising anode coating materials based on their significant reductive stability. On the cathode side, we single out MgF_2_, Mg(PO_3_)_2_, and MgP_4_O_11_ as effective passivating agents.

## Introduction

Multivalent batteries, such as those based on Mg, present a potential alternative to Li-ion batteries, particularly in terms of increased energy density (Canepa et al., 2017a). Mg batteries are able to use Mg metal as an anode at reasonable current densities (<0.5 mA/cm^2^) (Yoo et al., 2013), which in combination with the higher oxidation state of Mg (+2 rather than Li's +1) can provide a significant increase in the energy density of Mg batteries compared to Li-ion batteries. So far, prototypes of Mg batteries have utilized electrolytes, such as MgCl_2_ with AlCl_3_, Mg(ClO_4_)_2_, Mg(NO_3_)_2_, Mg(TFSI)_2_, and more complex molecules dissolved in acetonitrile, THF, or glymes-based solvents, in combination with Mg metal as the anode and a low voltage sulfide cathode (Mg_x_Mo_6_S_8_ and Mg_x_TiS_2_) (Aurbach et al., 2000; Cohen et al., 2000; Pour et al., 2011; Mohtadi et al., 2012; Muldoon et al., 2012, 2014; Yoo et al., 2013; Carter et al., 2014; Doe et al., 2014; Canepa et al., 2015a; Tutusaus et al., 2015, 2016; Sun et al., 2016; Hahn et al., 2018).

Typical Mg electrolytes have significantly narrower electrochemical stability windows (~1.5–3.0 V vs. Mg) (Lipson et al., 2016) compared to what is available in the Li-ion battery space (~1.5–5 V vs. Li) (Marom et al., 2011). Indeed, most electrolytes, including the solvents used in commercial Li electrolytes (e.g., PC and DMC) (Goodenough and Kim, 2009), have poor reductive stability (i.e., cathodic stability) and tend to decompose at the Mg metal anode (Lu et al., 1999; Muldoon et al., 2012). In addition, the utilization of high-voltage cathodes (e.g., oxides) is greatly impeded by the limited oxidative stability (i.e., anodic stability) of Mg electrolytes (Rosenberg and Nicolau, 1964; Cohen et al., 2000; Pour et al., 2011; Mohtadi et al., 2012; Muldoon et al., 2012, 2014; Yoo et al., 2013; Carter et al., 2014; Doe et al., 2014; Canepa et al., 2015a; Liu et al., 2015; Tutusaus et al., 2015, 2016; Chen et al., 2017; Hahn et al., 2018). Thus, the reactivity of the electrolyte against both Mg-anode and a high-voltage cathode results in electrolyte decomposition, often producing a passivating layer primarily containing a binary Mg-salt, such as MgO (and Mg(OH)_2_ if moisture is present) (Gofer et al., 2003; Ling et al., 2015; Ling and Zhang, 2017; Hannah et al., 2018). The presence of MgO greatly inhibits Mg^2+^ transport (Canepa et al., 2017b) and eventually the ability of the battery to store energy reversibly (Levi et al., 2009). Further work is still being done to develop Mg electrolytes that can reversibly strip and deposit Mg at the anode and cathode (Muldoon et al., 2012, 2014; Canepa et al., 2015b). For example, a class of carboranes has recently been proposed as promising electrolytes, stable against Mg metal and high voltage cathodes (up to 4.6 V vs. Mg) (Hahn et al., 2018). However, more work is required to elucidate the mechanisms of reversible Mg transfer at the cathode and develop strategies to mitigate electrolyte decomposition (Shao et al., 2013; Keyzer et al., 2016).

In analogous Li-systems, several approaches have been utilized to address the safety and electrochemical stability limitations of typical Li electrolytes (Aurbach et al., 2004; Guerfi et al., 2010). For example, solid electrolytes have been shown to be safer compared to typical solvent-based electrolytes, which may experience thermal runaway issues (Kamaya et al., 2011; Masquelier, 2011; Bachman et al., 2015; Kato et al., 2016). Another ongoing field of research is the application of protective coating layers to shield one or both electrodes from an incompatible electrolyte, while providing sufficient ionic mobility and preferably low electronic conductivity. Indeed, the solid electrolyte interphase (SEI) that forms at the graphitic anode-electrolyte interface is a good example of a protective layer with sufficient Li mobility that enables the reversible operation of Li-ion batteries (Verma et al., 2010). Therefore, similar solutions can be envisioned for Mg-batteries as well. To accomplish this goal, we searched for materials that can act as either protective coatings or even solid electrolytes by analyzing the electrochemical stability of various Mg-containing compounds.

Using a combination of density functional theory (DFT) calculations and thermodynamics, we assessed the electrochemical stability of various Mg-binary and ternary compounds, which may form as a result of electrolyte decomposition at either the Mg-metal anode or a high-voltage cathode. Specifically, we considered all Mg binaries and ternaries that do not contain redox-active metal ions (except Ti^4+^) and that are known to be electronic insulators. The choice of Mg compounds is also motivated by the highly reducing conditions that appear when in contact with Mg metal. For example, Li binaries and ternaries, such as Li_3_N, Li_3_P, LiH, Li_2_S, Li_2_O, and LiCl, tend to form (and be stable) at the Li electrolyte-anode interface in Li-ion batteries (Richards et al., 2015).

By calculating the electrochemical stability windows of candidate compounds, we identified their oxidative and reductive voltages. Our findings provide general guidelines for developing, via either *in situ* or *ex situ* deposition techniques, protective coating materials that are compatible with the anode or the cathode or both. Provided good bulk Mg^2+^ mobility exists (Sai Gautam et al., 2017), some of these materials may be investigated as protective coating materials or even solid electrolytes.

## Methodology

**Figure 1:**
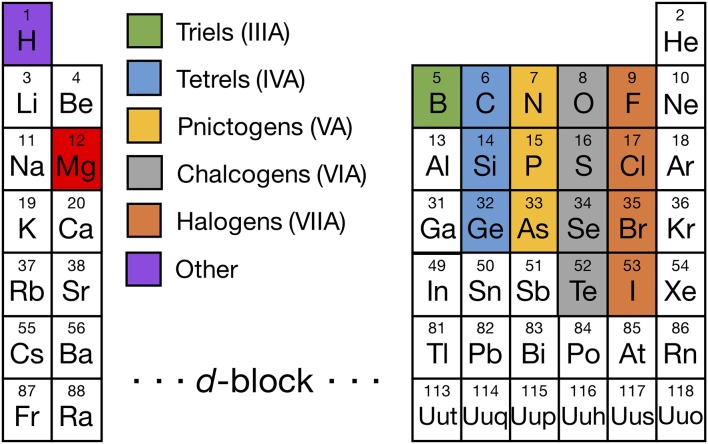
Periodic table highlighting the non-transition-metal elements that form binary (and ternary) compounds with Mg (red), including triels (Group IIIA, green), tetrels (Group IVA, light blue), pnictogens (Group VA, yellow), chalcogens (Group VIA, gray), halogens (Group VIIA, orange), and other elements (Hydrogen, purple). We considered all Mg-X binaries and stable Mg-X-Y ternaries, where X and Y are highlighted elements, with the exception of the Mg-X-H chemical space where only Mg-B-H compounds were considered. In addition, we evaluated some compounds containing a non-Mg metal, such as Sc, Ti, Nb, Zr, Al, Ga, and In, because either they are commonly used as coating materials in Li-ion batteries or have been considered as Mg ionic conductors in prior studies.

The set of elements from which we evaluated Mg binaries and ternaries is shown in Figure 1, with Mg colored in red and the other elements colored based on their respective group numbers (a complete list of all Mg-binaries and ternaries investigated is provided in Tables S1, S2). In addition to the highlighted elements, we considered borohydrides, niobates, titanates, titanium phosphates, and zirconium phosphates which have been reported to be promising coating materials in Li-ion batteries (Richards et al., 2015). Also, we included Mg-(Sc/In)-(S/Se) compounds since they have been explored as potential Mg solid-electrolyte materials in prior studies (Canepa et al., 2017b,c), apart from Mg-(Al/Ga/In)-(O/S/Se).

The electrochemical stability windows of each compound are calculated using the approach developed by (Richards et al., 2015) by constructing the corresponding grand potential (ϕ) phase diagram by means of the pymatgen library (Jain et al., 2011; Ong et al., 2013), where ϕ is defined as:

(1) $$\begin{array}{l} \phi \left[c,\;\mu_{Mg}\right]=\;E\left[c\right]-\;n_{Mg}\left[c\right]\times \mu_{Mg} \end{array}$$

For all μ_*Mg*_, we constructed the convex hull in the grand potential composition-space and identified compounds that are stable at each μ_*Mg*_. The Mg chemical potential μ_*Mg*_ relates directly to the voltage vs. Mg/Mg^2+^ via (Equation 2):

(2) $$\begin{array}{l} V=\;-\frac{\mu_{Mg}}{zF} \end{array}$$

where *F* is the Faraday constant, *z* is the number of electrons transferred (*z* = 2 for Mg) and μ_*Mg*_ is referenced to the energy of Mg metal. The internal energy of each compound [*E* in Equation (1)], in the relevant chemical space, was either obtained from the Materials Project (Jain et al., 2011, 2013) database or calculated directly using DFT (Kohn and Sham, 1965; Hohenberg and Kohn, 1973) (see Input parameters for DFT calculations in SI for more details on the calculation parameters used). For each compound, we utilized the atomic coordinates reported in the Inorganic Crystal Structure Database (ICSD) (Bergerhoff and Brown, 1987) as initial guesses during our DFT structure relaxation. For Mg_0.5_Zr_2_(PO_4_)_3_ and Mg_0.5_Ti_2_(PO_4_)_3_, which are disordered structures in the ICSD database, we enumerated possible configurations within the respective unit cell (Hart and Forcade, 2008, 2009; Hart et al., 2012; Ong et al., 2013) and included the lowest energy configuration.

## Results and Discussion

### Electrochemical Stability Windows of Mg-Binaries

**Figure 2:**
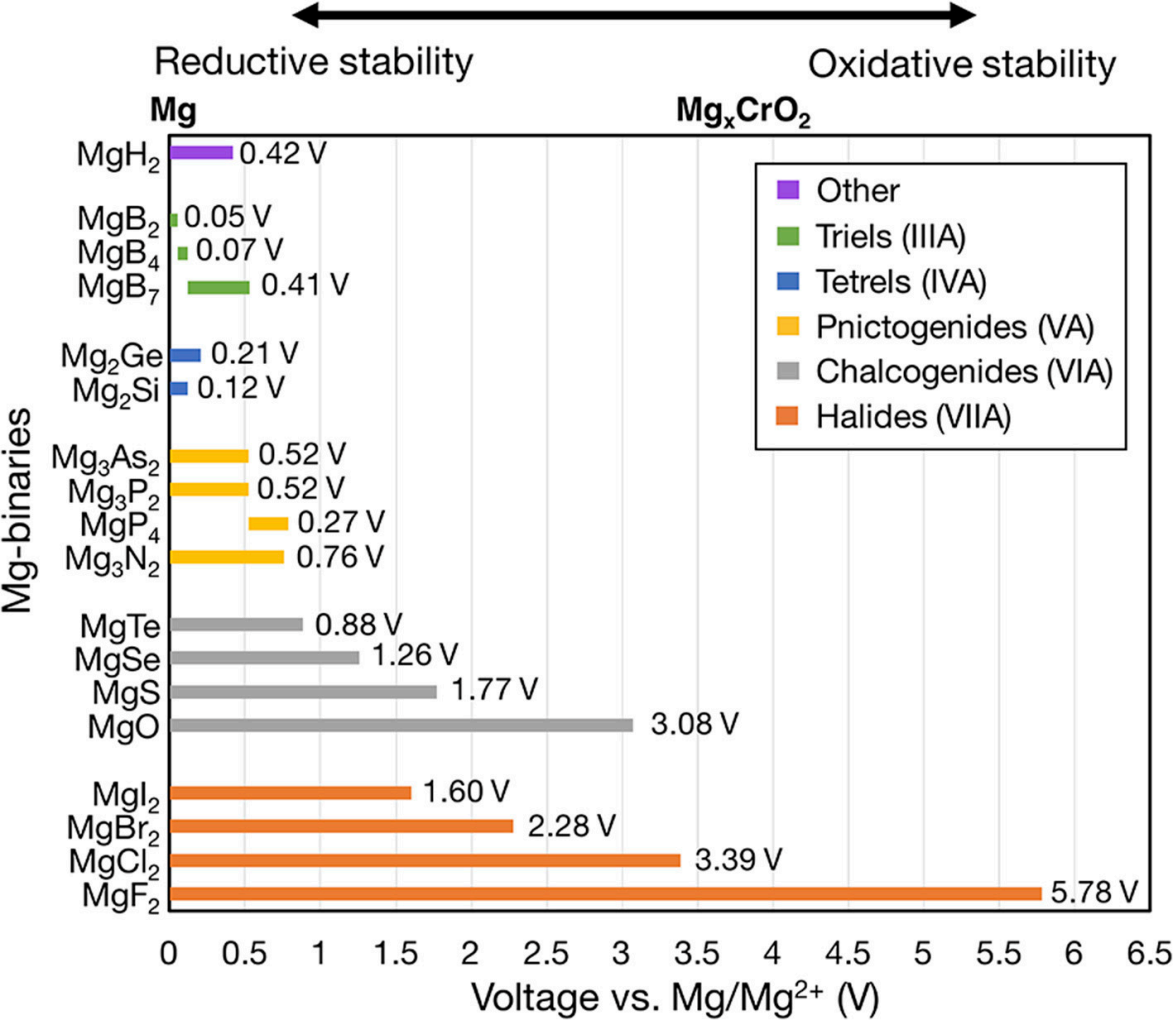
Electrochemical stability windows of non-metallic Mg-binaries, indicating the voltages (vs. Mg metal) at which the compound is stable against decomposition. Compounds that are not stable at any voltage, such as the Mg-carbides, are not shown. Compounds are grouped by the anion column and compounds within each group are ordered by increasing electronegativity. For systems with multiple compositions, compounds are ordered by decreasing ratio of Mg to anion. The number at the end of each bar indicates the width of the voltage window. The Mg_x_CrO_2_ spinel is shown above the plot at its calculated average voltage (~3.6 V) for reference.

Figure 2 shows the voltage windows of all Mg-X binaries considered, where the compounds are grouped by the anion column (Figure 1) and sorted within each group by increasing electronegativity.

To form a binary system with multiple stable compounds (e.g., Mg-B), we ordered the compounds according to a decreasing ratio of Mg to anion (Mg:B). Only binaries that were thermodynamically stable (i.e., with negative formation energy at 0 K) are shown. Unstable compounds have been removed from Figure 2 because they will not be stable at any μ_*Mg*_. For example, MgC_2_ has a formation energy of 173 meV/atom at 0 K. The left and right ends of the bar for each compound indicate the lower and upper voltage limits, corresponding to the reductive (cathodic) and oxidative (anodic) stabilities, respectively. Lower reductive stabilities and higher oxidative stabilities imply better resistance against reduction and oxidation, respectively. Thus, the width of the bar (text annotation to the right of each bar in Figure 2) for a given compound signifies its electrochemical stability window. The zero on the voltage axis is referenced to bulk Mg metal (i.e., V vs. Mg/Mg^2+^). Higher voltage values mimic the open circuit voltages of cathode materials, such as Chevrel-Mo_6_S_8_ (~1.1 V) (Aurbach et al., 2000), layered-V_2_O_5_ (~3.3 V) (Sai Gautam et al., 2015), or Mg_x_CrO_2_ (~3.6 V) (Chen et al., 2017).

Significantly, all of the Mg-halides, Mg-chalcogenides, and Mg-pnictides (except MgP_4_) are stable at 0 V vs. Mg/Mg^2+^ and thus stable against Mg metal. Among the Mg-triels and Mg-tetrels, only MgB_2_, Mg_2_Ge, and Mg_2_Si are stable vs. Mg metal. However, the widths of the stability windows of MgB_2_, Mg_2_Ge, Mg_2_Si are small (<0.1 V), and thus Mg-triels and Mg-tetrels do not appear to be viable coating materials against typical electrolytes. The poor stability windows of MgB_2_, and Mg_2_Ge, Mg_2_Si may be attributed to the weak electronegativity of the anions (i.e., B, Ge, and Si) and a consequent low resistance to oxidation. Additionally, B forms three thermodynamically stable compounds at various oxidation states with Mg, namely MgB_2_ (oxidation state of B is −1), MgB_4_ (B^−0.5^), and MgB_7_ (B^−0.28^). While MgB_2_ is stable against Mg metal (highest reducing conditions), at increasing voltages (~0.05 V vs. Mg/Mg^2+^), compounds with higher B oxidation states become stable, limiting the oxidative stability of MgB_2_. On the other hand, Cl and Mg only form MgCl_2_ as a stable binary, which oxidizes directly to Cl_2_ gas at ~3.39 V vs. Mg/Mg^2+^. Notably, MgCl_2_ is used as a precursor for Mg-Al-Cl-based electrolytes and its limited solubility in an ether-based solvent (typically used in Mg batteries) is well-documented (Doe et al., 2014; Canepa et al., 2015a). Therefore, MgCl_2_ may already be present in existing electrolytes, given its stability against Mg-metal (Figure 2), and may inherently protect the anode against further reactions with the electrolyte. In light of this, the role of MgCl_2_ as a potential protective coating layer on the Mg metal electrode needs to be further investigated.

Within each group of compounds of Figure 2 (i.e., each column of Figure 1), there is a strong correlation between the electronegativity of the anion and the oxidative stability. For example, within halogen compounds (orange bars), the oxidative stability rigorously follows the order MgF_2_ > MgCl_2_ > MgBr_2_ > MgI_2_, which correlates with the relative order of electronegativity of F > Cl > Br > I. Analogous trends can be observed among chalcogens (gray bars) and pnictogens (yellow). From this analysis we concluded that the electronegativity of the anion can be used as a proxy for the oxidative potential of Mg binary compounds since it describes the ability of the anion to limit an oxidation reaction.

### Electrochemical Stability Windows of Mg-Ternaries

**Figure 3:**
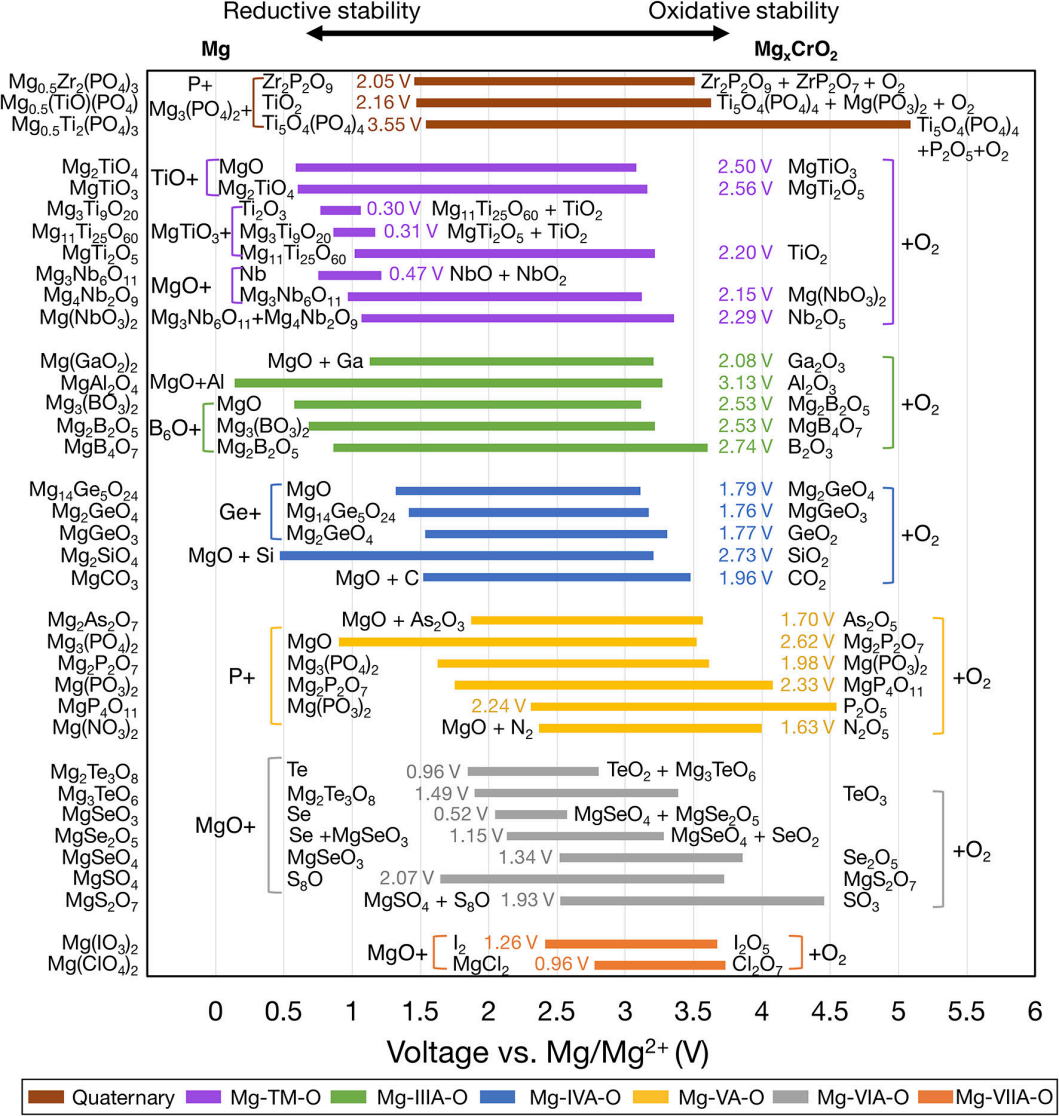
Electrochemical stability windows of Mg-ternary and quaternary oxides, indicating the voltages (vs. Mg metal) at which the compound is stable against decomposition. Compounds that are not stable at any voltage are not shown. Ternaries are grouped by the periodic table column of the non-Mg, non-anion elements and ordered within each group by increasing electronegativity of the non-Mg cation. For systems with multiple compositions, compounds are ordered by increasing reductive stability. The text next to each bar indicates the width of the voltage window and the decomposition products at the reductive and oxidative limits. Compounds sharing common decomposition products, such as MgO or O_2_ are grouped together by brackets.

**Figure 4:**
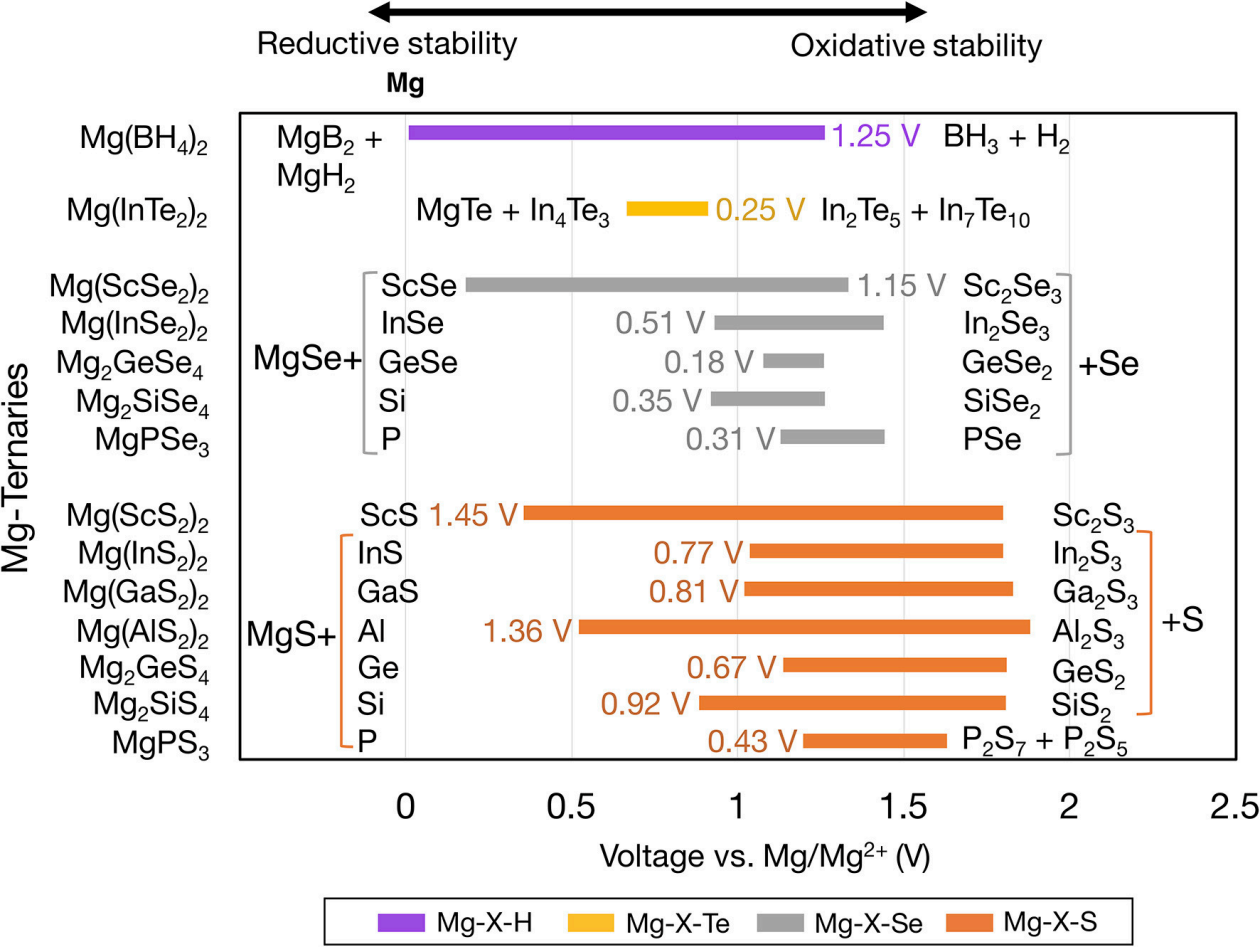
Electrochemical stability windows of Mg-ternary non-oxides, indicating the voltages (vs. Mg metal) at which the compound is stable against decomposition. Compounds that are not stable at any voltage are not shown. Ternaries are grouped by anion in order of increasing electronegativity and ordered within each group by increasing electronegativity of the non-Mg cation (e.g., P, S). The text next to each bar indicates the width of the voltage window and the decomposition products at the reductive and oxidative limits. Compounds sharing common decomposition products such as MgS or S are grouped together by brackets.

Figure 3 shows the voltage windows of Mg ternary and quaternary oxides, while Figure 4 shows the voltage windows of Mg ternary non-oxides (i.e., sulfides, selenides, tellurides, and a hydride).

The widths of the voltage windows are written next to the respective bars on either the left or the right side. Decomposition products at the reductive (oxidative) stability limits are written to the left (right) of the bars. For compounds within a group that share a common decomposition product (such as, MgO, O_2_ in Figure 3, and MgS, S or MgSe, Se in Figure 4), the common compounds are factored out and indicated in brackets. The compounds shown are based on the elements highlighted in Figure 1 and a set of stable Mg-niobates, Mg-titanates, Mg-titanium-phosphates and Mg-zirconium-phosphates are plotted vs. Mg/Mg^2+^ as the reference. Compounds that are not thermodynamically stable (i.e., with a non-zero decomposition energy or energy above the convex hull) are not plotted. For example, Mg_14_Si_5_O_24_ is calculated to decompose into Mg_2_SiO_4_ and MgO and hence not included in Figure 3. Of note, Mg does not form ternary halides where the halogen is the anion, according to the structures available in the ICSD. Thus, no compounds in the ternary phase spaces of Mg-P-Cl, Mg-N-F, Mg-B-F are known to exist. Instead, we found that the stable Mg-ternaries are ternary chalcogenides, where the anion is oxygen, sulfur, selenium, or tellurium (except for the Mg-borohydride).

Based on Figures 3, 4, we observed that Mg ternaries do not show reductive stability against Mg metal, as indicated by the lack of reductive stability down to 0 V for any compound considered. The ternary with the best reductive stability is Mg(BH_4_)_2_ (purple bar in Figure 4), which is stable up to 0.01 V against Mg metal. Further, none of the ternary compounds exceed the anodic stability of MgF_2_ (~5.8 V, Figure 2). Among the ternaries, the Mg-B-O-based compounds, Mg_2_SiO_4_ and Mg_3_(PO_4_)_2_ have the widest stability windows, with voltage window widths >2.5 V. Additionally, there exist a few ternary oxides, such as MgP_4_O_11_ (~4.55 V), MgS_2_O_7_ (~4.45 V), and a quaternary Mg_0.5_Ti_2_(PO_4_)_3_ (~3.82 V) which have significantly high oxidative stability and may represent potential protective coatings for high-voltage oxide cathodes (Chen et al., 2017).

In general, trends in oxidative and reductive stability from Figures 3, 4 can be explained by analyzing the species most prone to oxidation and reduction, respectively. In most of the ternary compounds considered, the species most prone to oxidation is the anion since the other elements are already at their highest oxidation states (e.g., P^5+^ in Mg_3_(PO_4_)_2_). Thus, the susceptibility of the anion to be oxidized dictates the oxidative stability of the compound. For example, among the phosphates (yellow bars in Figure 3), thio-phosphates (orange bar in Figure 4), and seleno-phosphates (gray bar in Figure 4), phosphates exhibit the highest oxidative stabilities compared to MgPS_3_ and MgPSe_3_ because O^2−^ is more difficult to oxidize than S^2−^ or Se^2−^. Given that the electronegativity of the anion directly corresponds to the tendency of the anion to attract electrons and its resistance to oxidation, there is a high degree of correlation between increasing anion electronegativity (e.g., O > S > Se) (Pauling, 1932) and higher oxidative stabilities of binary (Figure 2) and ternary (Figures 3, 4) oxides compared to sulfides and selenides, respectively. Additionally, the hybridization of the anion (e.g., between O^2−^ and P^5+^ in ${\text{PO}}_{4}^{3-}$ moieties) tends to stabilize it by lowering the energy of its electronic states, making the anion more difficult to oxidize. For example, binary MgO, where O^2−^ hardly hybridizes with Mg^2+^, oxidizes at ~3.10 V vs. Mg. On the other hand, most Mg-ternary oxides (including the phosphates) oxidize at higher voltages (i.e., exhibit superior oxidative stability) due to the hybridization of the O^2−^ by the non-Mg cation, such as P^5+^, S^6+^, etc.

The reductive stability of ternary compounds depends primarily on two key metrics: (i) the electronegativity of the species that undergoes reduction, which is the non-Mg cation in ternary compounds, and (ii) the electronegativity of the anion that does not undergo reduction but regulates the thermodynamic stability of the ternary compound vs. the corresponding binary compounds. Notably, reductive stability correlates inversely with the electronegativity of the non-Mg cation species, since larger electronegativities reflect higher attraction toward electrons and a higher propensity for reduction. For example, the reductive stability of ternary compounds (Figure 3) follows the order Mg-Cl-O (~2.78 V vs. Mg) < Mg-S-O (~1.65 V) < Mg-P-O (~0.9 V) < Mg-Si-O (~0.47 V) < Mg-Al-O (~0.14 V), which is the inverse of the electronegativity trends, namely Cl (3.16) > S (2.58) > P (2.19) > Si (1.90) > Al (1.61) (Pauling, 1932). In the case of quaternary systems, such as Mg-Ti-P-O and Mg-Zr-P-O, we predicted that P^5+^ reduces in preference to Ti^4+^ and Zr^4+^ (brown bars in Figure 3), which is consistent with the larger electronegativity of P (2.19) vs. Ti (1.54) and Zr (1.33).

Importantly, higher electronegativity of the anion results in poorer reductive stability of the ternary compound. For example, the reductive stability among Mg-Ge-, Mg-Sc-, Mg-In-ternary oxides follows Mg-Ge-O (~1.32 V) < Mg-Ge-S (~1.13 V) < Mg-Ge-Se (1.08 V), Mg-Sc-S (~0.36 V) < Mg-Sc-Se (0.18 V), and Mg-In-S (~1.04 V) < Mg-In-Se (~0.93 V) < Mg-In-Te (~0.67 V), respectively, consistent with the anion electronegativity trend (O > S > Se > Te). Note that higher anion electronegativity leads to more stable Mg-binary compounds, i.e., Mg-binaries with larger stability windows (Figure 2), which are common decomposition products under reducing conditions. A more stable Mg-binary reflects a larger thermodynamic driving force for reduction, as quantified by the corresponding formation energy (MgO ~ −3.06 eV/atom, MgS ~ −1.76 eV/atom, MgSe ~ −1.25 eV/atom, and MgTe ~ −0.87 eV/atom) (Jain et al., 2013), resulting in a lower reductive stability. Interestingly, the compound with the highest reductive stability, Mg(BH_4_)_2_, is composed of a low electronegative anion and a non-Mg cation, H (2.20) and B (2.04), respectively. Thus, minimizing the electronegativities of both the non-Mg-cations and the anions could be the key to discovering ternary compounds that are stable against Mg-metal.

Notable exceptions to the aforementioned trends in reductive stability vs. (non-Mg cation/anion) electronegativity can be observed across different chemistries in Figures 3, 4. For example, electronegativity of B (2.04) > Ga (1.81) > Al (1.61), but the reductive stability of Mg-Al-O (~0.14 V) > Mg-B-O (~0.58 V) > Mg-Ga-O (~1.13 V). Similar trends can be observed among Mg-IVA-O, and Mg-VA-O compounds (Figure 3). Such anomalies can be attributed to two factors that override non-Mg-cation electronegativity trends: (i) stability of Mg-(IIIA/IVA/VA) binaries (signifying the thermodynamic driving force to form decomposition products), and (ii) the relative position of the empty electronic states of IIIA/IVA/VA elements, as influenced by the extend of hybridization with oxygen (difficulty in reducing the ternary compound). For example, the highest oxidative stability of binary Mg-Al alloys [~0.06 V (Jain et al., 2013), not shown in Figure 2)] is lower than both Mg-B compounds (~0.53 V, Figure 2) and Mg-Ga alloys (~0.19 V, not shown). On the other hand, the significant hybridization of the electronic states of P with O likely pushes the empty (anti-bonding) P states to higher energy levels, making P difficult to reduce in ternary Mg-P-O, compared to As in Mg-As-O and N in Mg-N-O.

In the case of reductive stability vs. anion electronegativities, the stability of Mg-Al-O (~0.14 V) > Mg-Al-S (~0.52 V), and Mg-P-O (~0.9 V) > Mg-P-S (~1.20 V), despite the electronegativity of O > S is another notable exception. Here, the discrepancy can be attributed to the stability of Al-O and P-O bonds in comparison to Al-S and P-S bonds, as quantified by the formation energies (Al_2_O_3_ ~ −3.44 eV/atom, Al_2_S_3_ ~ −1.46 eV/atom and P_2_O_5_ ~ −2.46 eV/atom and P_2_S_5_ ~ −0.64 eV/atom) (Jain et al., 2013). The higher stability of Al-O and P-O bonds is possibly due to better hybridization of Al and P among the oxides vs. sulfides, respectively. Thus, despite MgO creating a larger thermodynamic driving force for reduction than MgS (as indicated by the stability windows in Figure 2), the lack of affinity for S from Al and P in Mg-Al-, and Mg-P-ternaries facilitates the reduction of Al^3+^ and P^4+/5+^, respectively, in the ternary sulfides compared to the oxides.

### Potential Candidate Materials

Based on the voltage windows of the Mg binaries, ternaries and quaternaries in Figures 2–4, we suggest potential coatings on both the Mg metal//Mg electrolyte and the Mg electrolyte//cathode interfaces. At the cathode interface, the oxidative stability should be high for candidate compounds. Among the binaries, only MgF_2_ has an oxidation limit above 4.0 V, whereas among the ternaries, including Mg(PO_3_)_2_, MgP_4_O_11_, Mg(NO_3_)_2_, and MgS_2_O_7_ show oxidation limits above 4.0 V. Note that among the candidate materials, those with the widest voltage windows should be given preference, which may enable compatibility with liquid electrolytes that are stable against Mg metal. Therefore, among the high-oxidation-limit compounds, MgF_2_, Mg(PO_3_)_2_, MgP_4_O_11_, and Mg_0.5_Ti_2_(PO_4_)_3_, which have the widest voltage windows (all > 2.0 V), should be considered the most promising candidate materials.

For the Mg metal//Mg electrolyte interface, the reductive stability of a candidate compound should ideally be ~0 V vs. Mg metal. In this context, Mg(BH_4_)_2_, with a reductive stability of ~0.01 V vs. Mg is a promising candidate for a protective anode coating. Previous experiments utilizing Mg(BH_4_)_2_-containing electrolytes have reported the formation of a Mg-conducting interphase layer against Mg-metal with an oxidative stability of 1.7 V vs. Mg, which is generally in accordance with our computational results (1.25 V vs. Mg) (Mohtadi et al., 2012; Arthur et al., 2017). The higher oxidative stability of Mg(BH_4_)_2_ observed in experiments (1.7 V vs. Mg) compared to in theory (1.25 V vs. Mg) could be due to kinetic stability, which is not accounted for in our calculations. Thus, Mg(BH_4_)_2_ should be further investigated as a protective coating on the Mg-metal anode. Additionally, in scenarios where the reductive stability is < ~0.5 V, such as MgAl_2_O_4_, Mg_2_SiO_4_ (Figure 3), Mg(ScS_2_)_2_, and Mg(ScSe_2_)_2_ (Figure 4), the compounds may exist in a metastable manner and may still be valid candidates. For example, in Li-ion batteries the solid electrolyte, garnet-Li_7_La_3_Zr_2_O_12_, has an estimated reductive stability of ~0.1 V vs. Li but has been shown to be metastable against Li metal (Richards et al., 2015; Ma et al., 2016). However, recent theoretical and experimental studies have shown that Mg(ScS_2_)_2_ and Mg(ScSe_2_)_2_ tend to decompose to binary MgS/MgSe and ScS/ScSe against Mg metal, ruling out any metastable existence (Canepa et al., 2017b,c). Another case to consider is when the Mg metal anode is replaced by Bi (or Sb or their alloys) as the reductive potential of the anode is shifted by up to ~+0.32 V vs. Mg metal (Arthur et al., 2012). In case these alternative anodes are used, several coating materials, such as MgAl_2_O_4_ or Mg(ScSe_2_)_2_, could be envisioned as potential coating materials. Nevertheless, changing the anode chemistry can not only change the overall energy density of the cell but also introduce additional over-potentials for Mg alloying at the anode. Notably, all binaries considered should be stable vs. Mg metal, except for MgP_4_, MgB_4_, and MgB_7_ (Figure 2), and are candidates for protective coatings at the anode//electrolyte interface. Specifically, Mg-halides, including MgF_2_, MgCl_2_, MgBr_2_, which have voltage windows wider than 2.0 V, should be considered as the most promising candidates.

A number of studies have suggested that the Cl^−^ in magnesium-aluminum-chloride-based electrolytes can protect the Mg-metal anode during Mg deposition via adsorption on the Mg-metal surface (Aurbach et al., 2002; Doe et al., 2014; Canepa et al., 2015a,b; See et al., 2015, 2017; Salama et al., 2017). Our results suggest that MgCl_2_ is stable against the highly reductive environment of Mg-metal, showing a wide stability window ~3.39 V. We speculate that a layer of MgCl_2_ may form *in situ* as a protective coating, which is further justified by the sparing solubility of this salt in ether-based solvents (Doe et al., 2014; Canepa et al., 2015a; Salama et al., 2017). Therefore, a careful experimental characterization of the Mg//electrolyte interface will shed light on the role of the speciation of Cl in the form of MgCl_2_ or as a free ion.

**Figure 5:**
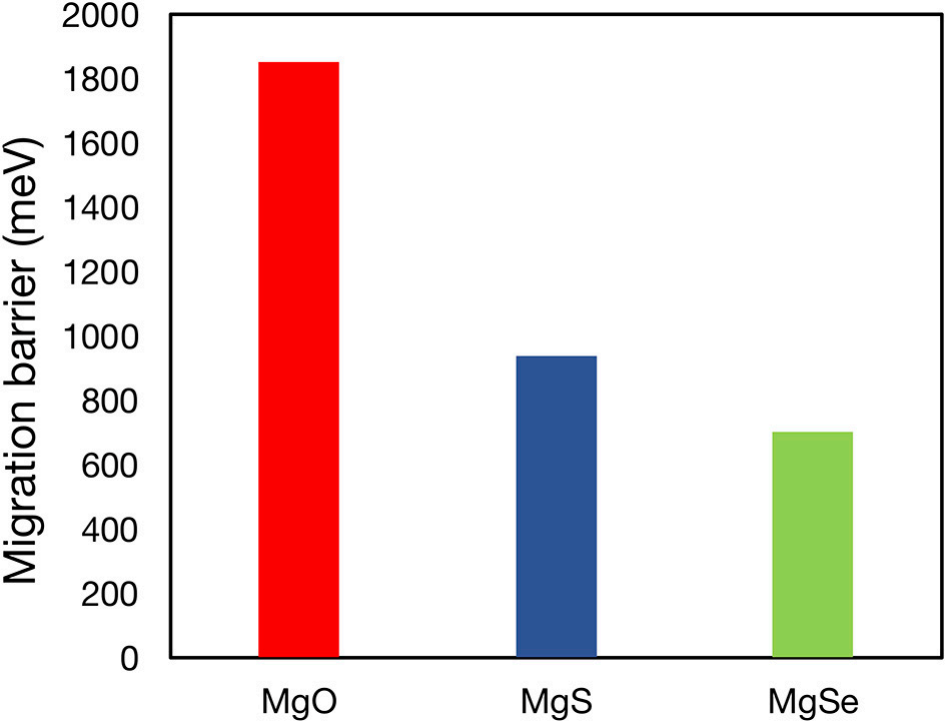
Plot of migration barriers of MgO (red), MgS (blue), and MgSe (green) as calculated in Canepa et al. (2017b). The high migration barriers of MgO, which is predicted to be stable vs. Mg metal and to have a reasonable oxidation limit (3.08 V vs. Mg metal), demonstrate the necessity of Mg^2+^ diffusivity data in determining the viability of potential coating and electrolyte materials.

For all of the suggested anode or cathode coating materials, a thorough evaluation of Mg^2+^ mobility is required to verify their viability as actual coating materials. Mobility evaluations are especially necessary to demonstrate proof-of-concept oxidative coatings that can enable high voltage cathodes [such as Mg_x_Cr_2_O_4_ (Chen et al., 2017), Mg_x_Mn_2_O_4_, (Sai Gautam et al., 2017), and Mg_x_V_2_O_5_ (Sai Gautam et al., 2015)] in conjunction with current liquid electrolytes and Mg-metal. Note that the Mg^2+^ migration barrier has been calculated for a number of Mg-binaries in a prior study (Canepa et al., 2017b), including MgO (~1,800 meV), MgS (~900 meV), and MgSe (~700 meV) of Figure 5, and a few ternaries, such as Mg(ScSe_2_)_2_ (~375 meV), Mg(InS_2_)_2_ (~488 meV), and Mg(ScS_2_)_2_ (~415 meV), while more studies are in progress for other candidates listed in this work. The poor bulk Mg mobility causes MgO and MgS to be inactive passivating materials that limit any Mg transference, despite their wide stability ranges (0–3.1 V for MgO and 0–1.6 V for MgS). Similarly, poor Mg mobility in bulk Mg_0.5_Ti_2_(PO_4_)_3_ [>1 eV (Canepa et al., 2017a)] will hinder its use as a protective oxidative coating. Nevertheless, our study identifies a tractable list of possible coating and electrolyte candidates in which Mg^2+^ mobility must be estimated, based on their calculated electrochemical stabilities.

## Conclusion

In this study, we evaluated, using density functional theory calculations, the electrochemical stability windows for non-redox-active Mg binary, ternary, and selected quaternary compounds in order to identify potential coating materials for Mg batteries. From the Mg binaries considered, we identified Mg-halides, specifically MgCl_2_ and MgBr_2_, as potential anode coating materials based on their reductive stability (at 0 V vs. Mg/Mg^2+^). We also suggested Mg(BH_4_)_2_, MgAl_2_O_4_, and Mg_2_SiO_4_, as possible ternary anode coating materials, given their reductive stability below 0.5 V, with MgAl_2_O_4_ and Mg_2_SiO_4_ exhibiting a voltage window that is >2.0 V wide. Additionally, we expect MgF_2_, Mg(PO_3_)_2_, and MgP_4_O_11_ to be promising candidates for protecting high-voltage cathodes against typical Mg electrolytes. While careful evaluation of Mg mobility in candidate materials is essential, this work identifies specific chemistries as well as general guidelines on compound stabilities that will be useful to design practical coating materials in Mg batteries.

## Author Contributions

PC and GS conceived the project. TC, GS, and PC performed the simulation and analyzed the data. TC, GS, and PC wrote the manuscript and discussed it with GC.

### Conflict of Interest Statement

The authors declare that the research was conducted in the absence of any commercial or financial relationships that could be construed as a potential conflict of interest.
