# Supplementary material for: Evaluation of Mg Compounds as Coating Materials in Mg Batteries
Source: Front Chem. 2019 Jan 30;7:24. doi: 10.3389/fchem.2019.00024 (PMC6363690; doi:10.3389/fchem.2019.00024)
Supplement: Supplementary file 1 [file Data_Sheet_1.PDF]

Evaluation of Mg compounds as coating materials in Mg batteries

Tina Chen,[1,2] Gerbrand Ceder,[1,2] Gopalakrishnan Sai Gautam,[3,#] and Pieremanuele Canepa[4,*]

[1]Department of Materials Science and Engineering, University of California Berkeley, California 94720, United States.
[2]Materials Science Division, Lawrence Berkeley National Laboratory, Berkeley, California 94720, United States.
[3]Department of Mechanical and Aerospace Engineering, Princeton University, Princeton, New Jersey 08544, United States.
[4]Department of Materials Science and Engineering, National University of Singapore, Singapore 117575, Singapore.

[#]Corresponding author: gautam91@princeton.edu
*Corresponding author: pcanepa@nus.edu.sg

S1. Mg-binary and ternary compounds

Table S1: List of the specific Mg-binaries whose reductive and oxidative stabilities have been evaluated.

**Mg-X**	**Binaries**
Mg-B	MgB_2
	MgB_4
	MgB_7
Mg-Si	Mg_2Si
	Mg_9Si_5
	Mg_5Si_6
Mg-C	Mg_2C_3
	MgC_2
Mg-As	Mg_3As_2
Mg-P	Mg_3P_2
	MgP_4
Mg-N	Mg_3N_2
Mg-Te	MgTe
Mg-Se	MgSe
Mg-S	MgS
Mg-O	MgO
Mg-I	MgI_2
Mg-Br	$MgBr_2$
Mg-Cl	$MgCl_2$
Mg-F	MgF_2
Mg-H	MgH_2

Table S2: List of the specific Mg-ternaries whose reductive and oxidative stabilities have been evaluated.

**Mg-X-Y**	**Ternaries**
Mg-In-Te	$Mg(InTe_2)_2$
Mg-Si-Se	Mg_2SiSe_4
Mg-P-Se	$MgPSe_3$
Mg-Sc-Se	$Mg(ScSe_2)_2$
Mg-In-Se	$Mg(InSe_2)_2$
Mg-Ge-Se	Mg_2GeSe_4
Mg-Si-S	Mg_2SiS_4
Mg-Ge-S	Mg_2GeS_4
Mg-P-S	$MgPS_3$
Mg-Al-S	$Mg(AlS_2)_2$
Mg-Ga-S	$Mg(GaS_2)_2$
Mg-Sc-S	$Mg(ScS_2)_2$
Mg-In-S	$Mg(InS_2)_2$
Mg-B-H	$Mg(BH_4)_2$
**Ternary oxides**	
Mg-B-O	$Mg_3(BO_3)_2$
	$Mg_2B_2O_5$
	$MgO(B_2O_3)_2$
Mg-Si-O	$Mg_{14}Si_5O_{24}$
	Mg_2SiO_4
	$MgSiO_3$
Mg-C-O	$MgCO_3$
Mg-As-O	$Mg_2As_2O_7$
Mg-P-O	$Mg_3(PO_4)_2$
	$Mg_2P_2O_7$
	$Mg(PO_3)_2$
	MgP_4O_{11}
Mg-N-O	$Mg(NO_3)_2$
Mg-Te-O	Mg_3TeO_6
	$Mg_2Te_3O_8$
	$MgTe_6O_{13}$
Mg-Se-O	$MgSeO_3$
	$MgSeO_4$
	$MgSe_2O_5$
Mg-S-O	$MgSO_4$
	MgS_2O_7
Mg-I-O	$Mg(IO_3)_2$
Mg-Cl-O	$Mg(ClO_4)_2$
Mg-Ti-O	Mg_2TiO_4
	$MgTiO_3$
	$Mg_3Ti_9O_{20}$
	$Mg_{11}Ti_{25}O_{60}$
	$MgTi_2O_5$
Mg-Nb-O	$Mg_3Nb_6O_{11}$
	$Mg_4Nb_2O_9$
	$MgNb_2O_6$
Mg-Ga-O	$MgGa_2O_4$

Mg-Al-O	$MgAl_2O_4$
Mg-Ge-O	$Mg_{14}Ge_5O_{24}$
	Mg_2GeO_4
	$MgGeO_3$

S2. Input parameters for DFT calculations

For compounds not available in the Materials Project database[1], we perform density functional theory (DFT)[2, 3] calculations as implemented in the Vienna Ab initio Simulation Package (VASP).[4,5] We use the Perdew-Burke-Ernzerhof (PBE) parametrization of the generalized gradient approximation (GGA) to describe the electronic exchange and correlation.[6] The wave functions of the valence electrons are expanded with a plane-wave basis using a well-converged energy cutoff of 520 eV, whereas the core electrons are treated with the projector augmented wave (PAW) scheme.[7] The Brillouin-zone integration is carried out on a Monkhorst-Pack[8] *k*-point mesh with reciprocal density of 64 *k*-points per $Å^{-1}$. The total energy is converged within $5*10^{-5}$ eV/atom.

References

1. Jain, A.; Ong, S. P.; Hautier, G.; Chen, W.; Richards, W. D.; Dacek, S.; Cholia, S.; Gunter, D.; Skinner, D.; Ceder, G., Commentary: The Materials Project: A materials genome approach to accelerating materials innovation. *Apl Materials* **2013,** 1, (1), 011002.
2. Kohn, W.; Sham, L. J., Self-consistent equations including exchange and correlation effects. *Physical Review* **1965,** 140, (4A).
3. Hohenberg, P., Kohn, W., Inhomogeneous electron gas. *Physical Review B* **1973,** 7, (5), 1912-1919.
4. Kresse, G.; Hafner, J., Ab initio molecular dynamics for liquid metals. *Physical Review B* **1993,** 47, (1), 558-561.
5. Kresse, G.; Furthmüller, J., Efficient iterative schemes for *ab initio* total-energy calculations using a plane-wave basis set. *Physical Review B* **1996,** 54, (16), 11169-11186.
6. Perdew, J. P.; Burke, K.; Ernzerhof, M., Generalized Gradient Approximation Made Simple. *Physical Review Letters* **1996,** 77, (18), 3865-3868.
7. Kresse, G.; Joubert, D., From ultrasoft pseudopotentials to the projector augmented-wave method. *Physical Review B* **1999,** 59, (3), 1758-1775.
8. Monkhorst, H. J.; Pack, J. D., "Special points for Brillouin-zone integrations" - a reply. *Physical Review B* **1977,** 16, (4), 1748-1749.
